# Commissioning and verification of a 3D Monte Carlo independent calculation software for O‐ring linac systems

**DOI:** 10.1002/acm2.70445

**Published:** 2025-12-29

**Authors:** Xiangyin Meng, Tingting Dong, Yongguang Liang, Zhen Zhou, Zhiqun Wang, Jie Qiu, Jingjing Zhao, Zheqing Zhang, Wenbo Li, Bo Yang

**Affiliations:** ^1^ Department of Radiation Oncology Peking Union Medical College Hospital Beijing China; ^2^ LAP Laser Applications China Co., Ltd. Shanghai China

**Keywords:** independent calculation, Monte Carlo, O‐ring linac, triangular gamma analysis

## Abstract

**Background:**

O‐ring linac systems improve radiotherapy efficiency but require rigorous pretreatment verification due to increased delivery uncertainty in IMRT/VMAT. Existing methods face limitations: measurement‐based approaches incur setup errors, while calculation‐based methods (e.g., Monte Carlo) need machine‐specific validation per AAPM TG‐219.

**Purpose:**

To commission the O‐ring linac model in RadCalc Monte Carlo software and establish its clinical dosimetric accuracy.

**Methods:**

The additional radiation to light field offset (ARLFO) parameter in RadCalc was adjusted from −0.08 cm (−0.8 mm) to +0.08 cm (+0.8 mm) (nine sets). TG‐119 and clinical benchmark cases were used to design IMRT/VMAT plans. Verification plans were measured experimentally, and all plans were imported into RadCalc for secondary dose calculation. Triangular gamma analysis (3%/2 mm) compared Monte Carlo‐simulated, measured, and TPS‐calculated doses. The optimal ARLFO was determined by weighted averaging of gamma pass rates. The model was validated on 10 clinical cases per site (head, thorax, abdomen, pelvis).

**Results:**

Commissioning identified the optimal ARLFO parameter as −0.02 cm (−0.02 mm). Under the gamma analysis criterion of 3%/2 mm, the comparison between TPS‐calculated doses and Monte Carlo‐calculated doses for 10 clinical plans across four anatomical sites yielded: 98.9% ± 0.8% (head and neck), 98.5% ± 0.8% (thorax), 99.6% ± 0.3% (abdomen), and 99.1% ± 0.5% (pelvis). For verification plans, the gamma pass rates between Monte Carlo‐calculated doses and measured doses were 97.5% ± 2.8% (head and neck), 95.6% ± 2.8% (thorax), 96.3% ± 2.9% (abdomen), and 99.4% ± 0.5% (pelvis), while comparisons of Monte Carlo‐calculated doses versus TPS‐calculated doses reached 99.5% ± 0.8% (head and neck), 99.2% ± 1.0% (thorax), 99.5% ± 0.9% (abdomen), and 99.9% ± 0.2% (pelvis), demonstrating consistent dosimetric accuracy of the optimized model across all clinical sites.

**Conclusion:**

This study establishes a commissioning methodology to determine the optimal ARLFO value for RadCalc, enabling clinics to achieve reliable independent plan verification for O‐ring linac.

## 1 INTRODUCTION

Advances in radiation therapy have yielded diverse radiotherapy systems with distinct functionalities. O‐ring gantry linear accelerators, such as Halcyon/Ethos (Varian Medical Systems, Palo Alto, CA, USA), are increasingly being adopted clinically because of their enhanced treatment efficiency, patient safety, comfort, and integrated design.^1^, ^2^, ^3^, ^4^, ^5^, ^6^ Although intensity‐modulated radiation therapy (IMRT) and volumetric modulated arc therapy (VMAT) improve target conformity and organ‐at‐risk sparing compared with 3D conformal radiotherapy (3D‐CRT), their increased complexity heightens delivery uncertainty.^7^, ^8^ This elevates the monitor units (MU), beam‐on time, leakage radiation, and integral dose, necessitating pretreatment verification. Concurrently, adaptive radiotherapy (ART) has been increasingly implemented in recent years. Commercial O‐ring linac platforms are available that provide either magnetic resonance imaging (MRI)‐guided ART^9^, ^10^ or cone‐beam computed tomography (CBCT)‐guided ART.^11^, ^12^, ^13^, ^14^ Each treatment session requires verification of the adaptive plan before delivery. Current verification methods face inherent limitations: measurement‐based approaches (using ion chambers, films, or 3D detectors) require phantom plan transfer introducing setup errors and fail to model patient heterogeneity while consuming substantial resources^15^, ^16^, ^17^; independent calculation‐based methods recompute 3D doses on patient CT using secondary algorithms (Monte Carlo, collapsed cone convolution superposition, or kernel‐based) such as RadCalc v7.4.1 (LAP GmbH, Lüneburg, Germany) with log files/EPID data^18^, ^19^, ^20^, ^21^ According to the AAPM Task Group Report 219 (TG‐219)^22^ institutional commissioning of such software is mandatory. Even with ‘golden’ beam data, machine‐specific variations require local validation. This study optimized the O‐ring linac parameters in RadCalc using TG‐119 test cases^23^ and institutional benchmark test cases, comparing Monte Carlo‐simulated, TPS (treatment planning system)‐calculated, and measured doses to establish the clinical accuracy across diverse anatomical sites.

## 2 MATERIAL AND METHODS

### 2.1 Monte Carlo independent calculation software

RadCalc utilizes a Monte Carlo algorithm based on BEAMnrc, enabling the precise modeling of multi‐leaf collimator (MLC) leaf trajectories and energy attenuation characteristics in linear accelerators. The software provides a fully automated pipeline encompassing TPS data import, simulation computation, and report generation along with functionalities for dose volume histogram (DVH) comparison, gamma analysis, and visualization of isodose distributions.

The O‐ring linac Halcyon utilizes preloaded representative beam data, which eliminates the need for physicists to collect treatment planning system (TPS) modeling data and enhance machine consistency across different institutions^24^ RadCalc incorporates a built‐in Halcyon beam model and provides the additional radiation to light field offset (ARLFO) parameter to fine‐tune the Monte Carlo algorithm outputs, enabling centers to establish clinically relevant models. This parameter (analogous to the dosimetric leaf gap (DLG) in TPS) defines the difference between the physical position of the MLC and its effective dosimetric position.^25^, ^26^ However, the methodology for determining ARLFO, as suggested by the manual, is not a direct measurement like the DLG. Instead, it is an offset value determined iteratively by comparing RadCalc's calculations against measured data until the best agreement is achieved. The ARLFO typically ranges from −0.1 cm (−1.0 mm) to +0.1 cm (+1.0 mm). A larger value indicates a greater distance between the MLC leaf ends in the Monte Carlo model. To determine a value that best fits our machine, we performed an iterative process across an adjustment range spanning from −0.08 cm (−0.8 mm) to +0.08 cm (+0.8 mm) with a step size of 0.02 cm (0.2 mm), resulting in nine independent computational models for evaluation.

The Monte Carlo simulations were performed using RadCalc version 7.4.1. The calculations were configured via the software's graphical interface with the following key transport settings to ensure an optimal balance between computational accuracy and efficiency. The statistical uncertainty in high‐dose regions (dose > 50% of maximum) was constrained to below 2.5%. Particle transport was governed by the default cut‐off energies of the underlying EGSnrc/BEAMnrc Monte Carlo code, set at electron cutoff energy (ECUT) = 700 keV and photon cutoff energy (PCUT) = 10 keV for photons. Dose was calculated on a 0.3 cm grid as dose‐to‐medium in heterogeneous mode, which incorporates density corrections derived from the patient's CT scan to account for tissue inhomogeneities. Under this configuration, the typical computation time per plan ranged from 2 to 5 minutes on a standard clinical workstation.

### 2.2 TG‐119 test cases

Prior to the plan design, simulation scans were performed using the Philips Big Bore CT (Philips Healthcare, Amsterdam, Netherlands) with a tube voltage of 120 kV, tube current of 220 mA, and slice thickness of 2.5 mm. The acquired images were imported into Eclipse TPS v15.6 (Varian Medical Systems, Palo Alto, CA, USA). Based on the requirements of different test cases, the target volumes and organs at risk (OARs) were contoured. For instance, in a prostate benchmark test case, structures include the planning target volume (PTV), bladder, rectum, and femoral head. Both the VMAT and IMRT plans were designed for each test case owing to their distinct technical characteristics. The modulation complexity of each plan was quantified using the modulation complexity score (MCS).^27^ Specifically, to address the dual‐layer MLC system of the Halcyon accelerator, the MCS values were first determined separately for the proximal (MCS‐P) and distal (MCS‐D) banks. Subsequently, these two values were averaged to obtain a single, representative MCS value for the plan. For every base plan, a corresponding ArcCHECK (Sun Nuclear Corporation, Melbourne, FL, USA) verification plan is generated. All dose calculations in the TPS employ version 15.6 of the anisotropic analytical algorithm (AAA).

Given the substantial volume of experimental data from the nine test models, this subset of cases is designated for pre‐commissioning, with the aim of selecting two optimal models via weighted ensemble evaluation of their performance on TG‐119 test cases, thereby narrowing the scope for subsequent formal validation.

### 2.3 Benchmark test cases

The benchmark test cases comprised 11 clinically selected disease types (cervical cancer, rectal cancer, gastric cancer, hepatocellular carcinoma, and lung cancer, as detailed in Table 1). These test cases were utilized in our center for TPS commissioning and post‐upgrade validation of diverse algorithms. As emphasized in the TG‐219 Report, patient CT images and anatomical structures provide superior simulation of tissue heterogeneity compared to homogeneous phantoms.

**TABLE 1:** Patient characteristics of benchmark test cases.

Patient number	Diagnosis
1	Cervical cancer
2	Rectal adenocarcinoma
3	Gastric adenocarcinoma
4	Cervical cancer (with lymph node metastasis)
5	Hepatocellular carcinoma
6	Lung cancer
7	Lung cancer (stereotactic body radiotherapy)
8	Pancreatic ductal adenocarcinoma
9	Esophageal squamous cell carcinoma
10	Thymoma
11	Oropharyngeal squamous cell carcinoma
12	Nasopharyngeal carcinoma
13	Glioblastoma
14	Metastatic brain carcinoma

Mirroring the pre‐commissioning workflow for the TG‐119 test cases, both VMAT and IMRT plans were designed for each benchmark case, and a corresponding ArcCHECK verification plan was generated for each base plan.

This cohort served for fine‐tuning, enabling the selection of the optimal model from two candidate parameter sets as the final commissioning outcome.

### 2.4 Clinical test cases

The clinical test cases comprised 40 patients (10 cases per anatomical site: head‐neck, thorax, abdomen, and pelvis) randomly selected from those treated with the Halcyon accelerator between January and April 2025. The inclusion criteria mandated that all selected cases must have previously passed our institutional patient‐specific quality assurance protocol and have complete datasets available, including planning CT images, structure sets, treatment plans, and corresponding ArcCHECK measurement data. Cases were excluded if they were treated with techniques other than VMAT or IMRT, had incomplete data, or had failed the initial patient‐specific QA. This selection strategy was designed to provide a robust validation dataset that reflects a typical clinical workflow while ensuring data integrity for the independent dose calculation model validation.

ArcCHECK verification plans were designed based on the original plans. Both the base and verification plans were imported into RadCalc for the secondary dose calculation using the Monte Carlo method. This cohort was used to validate the accuracy of the optimal dose calculation model in clinical scenarios.

### 2.5 Triangular gamma analysis

The institutional practices for secondary dose verification software may vary. Some institutions perform independent verification by importing original treatment plans, CT images, and dose data from TPS into the software, whereas others import phantom‐based verification plans, images, and doses to reduce the workload of physical measurements.^28^ Consequently, the Monte Carlo simulation results should be consistent with both the TPS calculations and physical measurements.

**FIGURE 1:**
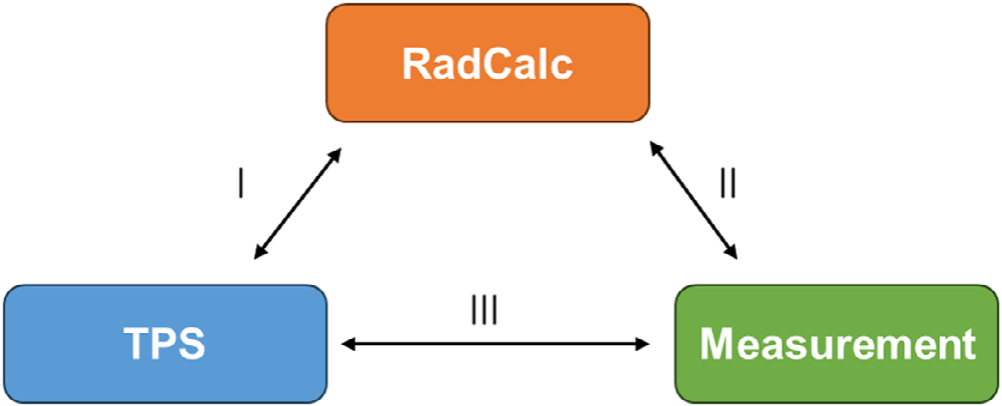
Flowchart of the triangular gamma analysis methodology. This diagram illustrates the three‐way comparison framework used in this study, detailing the data sources and analysis pathways between the treatment planning system (TPS), RadCalc Monte Carlo simulation, and ArcCHECK measurement.

Therefore, A tripartite dose comparison among the RadCalc Monte Carlo‐simulated doses, ArcCHECK‐measured doses, and TPS‐calculated doses was performed to resolve systematic discrepancies. This ‘triangular gamma analysis’ workflow, detailed in Figure 1, was implemented as follows, utilizing the standard gamma analysis modes of each software:

- RadCalc vs. TPS (3D Analysis): The original treatment plan was imported from the TPS into RadCalc (v7.4.1). After the Monte Carlo simulation, a 3D global gamma analysis was performed within the RadCalc software, using the TPS‐calculated dose as the reference dataset.
- RadCalc vs. Measurement (2D Analysis): The verification plan was imported from the TPS into RadCalc for Monte Carlo simulation. The resulting calculated dose distribution was exported to SNC Patient software v8.0 (Sun Nuclear Corporation, Melbourne, FL, USA). A 2D global gamma analysis was performed within SNC Patient, comparing it against the measured dose from ArcCHECK (reference).
- TPS vs. Measurement (2D Analysis): The verification plan was imported from the TPS directly into SNC Patient software v8.0. A 2D global gamma analysis was performed, comparing the TPS‐calculated dose against the measured dose (reference).

All gamma analyses strictly adhered to the AAPM TG‐218 Report^15^ employing unified parameters: 3%/2 mm criteria (distance‐to‐agreement/dose difference), 10% dose threshold (relative to maximum dose), and absolute dose normalization in global mode, with a gamma pass rate (GPR) > 90% required for clinical acceptance.

The results of this three‐way comparison were used to select the optimal ARLFO parameter based on a composite decision rule. A value was deemed clinically acceptable only if it simultaneously achieved high GPR in all three pairwise comparisons (RadCalc vs. TPS, RadCalc vs. Measurement, TPS vs. Measurement). This stringent criterion ensured that the final selected parameter produced results that were consistent with the TPS, accurate against measurement, and based on a valid delivery baseline.

## 3 RESULTS

### 3.1 TG‐119 test cases

#### 3.1.1 VMAT results

**FIGURE 2:**
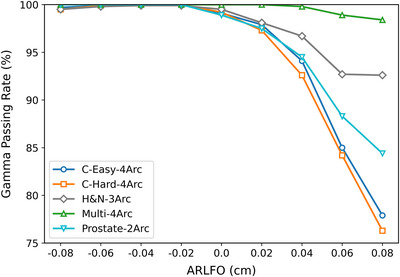
Gamma pass rates (%) between RadCalc Monte Carlo‐simulated doses and TPS‐calculated doses corresponding to different ARLFO values in VMAT technique for TG‐119 test cases.

Gamma analysis comparing RadCalc and TPS dose distributions revealed that the GPR approached 100% for both systems when ARLFO was ≤0 cm. However, as the ARLFO increased beyond 0, the GPR values exhibited a monotonic decline (Figure 2 and Table 2). As illustrated in Table 3, further analysis revealed that the extent of this decline was strongly correlated with the modulation complexity of the plans. The three plans with the highest modulation complexity (lowest MCS values)—Prostate‐2Arc (MCS = 0.07), C‐Hard‐4Arc (MCS = 0.11) and C‐Easy‐4Arc (MCS = 0.12)—exhibited the most substantial decrease in GPR. Among these, the C‐Hard‐4Arc plan demonstrated the steepest decline, indicating the highest sensitivity to inaccuracies in the MLC modeling parameter. Conversely, the two plans with the lowest complexity (highest MCS values)—H&N‐3Arc (MCS = 0.29) and Multi‐4Arc (MCS = 0.17)—showed a markedly more stable and robust response to ARLFO variations. This clear grouping indicates that plans with higher modulation complexity are significantly more susceptible to errors in the ARLFO parameter, while simpler plans are more forgiving. Based on weighted average GPR, we identified ARLFO = −0.04 cm (−0.4 mm) and −0.02 c (−0.2 mm)m as candidate values for clinical implementation.

**TABLE 2:** Gamma pass rates (%) between RadCalc and TPS‐calculated doses for TG‐119 test cases using VMAT.

ARLFO (cm)	C‐Easy‐4Arc	C‐Hard‐4Arc	H&N‐3Arc	Multi‐4Arc	Prostate‐2Arc	Average
−0.08	99.7	99.5	99.5	100.0	100.0	99.7 ± 0.2
−0.06	99.9	99.9	99.8	100.0	100.0	99.9 ± 0.1
−0.04	100.0	100.0	99.9	100.0	100.0	100 ± 0
−0.02	100.0	100.0	99.9	100.0	100.0	100 ± 0
0	99.1	99.2	99.5	100.0	98.9	99.3 ± 0.4
0.02	97.9	97.3	98.1	100.0	97.5	98.1 ± 1.1
0.04	94.1	92.6	96.7	99.8	94.5	95.6 ± 2.8
0.06	85.0	84.2	92.7	98.9	88.3	89.8 ± 6.1
0.08	77.9	76.3	92.6	98.4	84.4	85.9 ± 9.5

**TABLE 3:** MCS values for the TG‐119 test cases.

Technique	Plans	MCS‐D	MCS‐P	Averaged‐MCS
VMAT	C‐Easy‐4Arc	0.10	0.14	0.12
	C‐Hard‐4Arc	0.09	0.12	0.11
	H&N‐3Arc	0.28	0.30	0.29
	Multi‐4Arc	0.16	0.18	0.17
	Prostate‐2Arc	0.06	0.08	0.07
IMRT	C‐Easy‐9F	0.04	0.04	0.04
	C‐Hard‐9F	0.03	0.03	0.03
	H&N‐9F	0.05	0.05	0.05
	Multi‐7F	0.13	0.13	0.13
	Prostate‐7F	0.03	0.03	0.03

**FIGURE 3:**
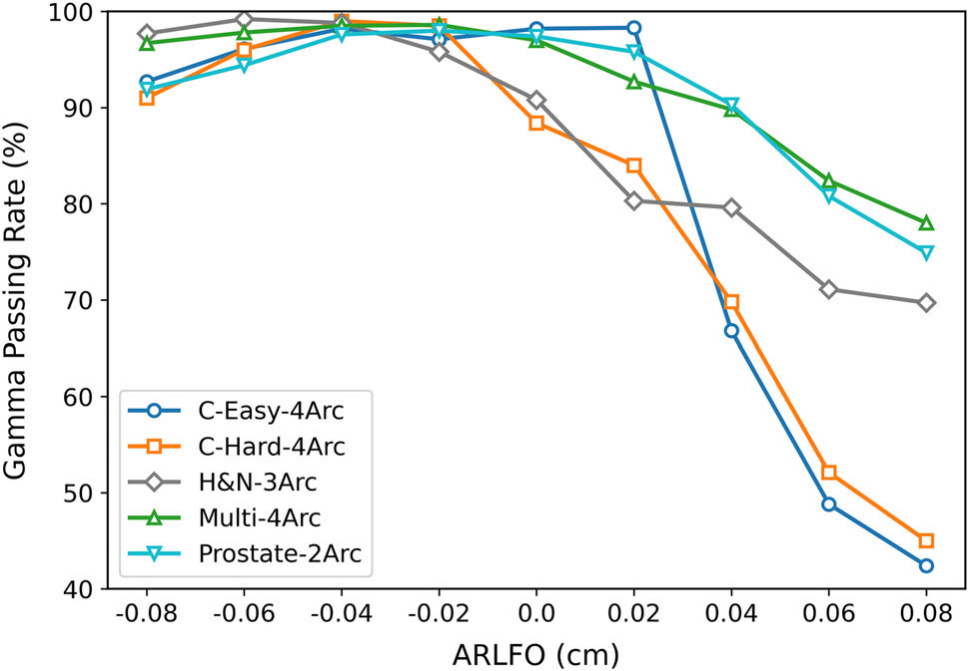
Gamma pass rates (%) between RadCalc Monte Carlo‐simulated doses and ArcCHECK‐measured doses corresponding to different ARLFO values in VMAT technique for TG‐119 test cases.

Our gamma analysis of dose distributions between RadCalc‐calculated and ArcCHECK‐measured data revealed a nonmonotonic relationship between ARLFO values and GPR. As shown in Figure 3 and Table 4, for ARLFO < 0 cm, GPR initially increased and then decreased as ARLFO decreased, reaching a peak at ARLFO = −0.04 cm (maximum weighted average GPR: 98.4% ± 0.5% under the 3%/2 mm criterion). For ARLFO > 0 cm, the GPR exhibited a monotonic decline with increasing ARLFO values. Based on weighted average GPR, we identified ARLFO = −0.04 cm (−0.4 mm) and −0.02 (−0.2 mm) cm as optimal candidates. This result perfectly aligns with the prior gamma analysis between the RadCalc‐calculated and TPS‐calculated doses.

**TABLE 4:** Gamma pass rates (%) between RadCalc and ArcCHECK‐measured doses for TG‐119 test cases using VMAT.

ARLFO (cm)	C‐Easy‐4Arc	C‐Hard‐4Arc	H&N‐3Arc	Multi‐4Arc	Prostate‐2Arc	Average
−0.08	92.7	91	97.7	96.7	91.9	94.0 ± 3.0
−0.06	96.1	96	99.2	97.8	94.4	96.7 ± 1.8
−0.04	98.2	99	98.8	98.5	97.6	98.4 ± 0.5
−0.02	97.1	98.5	95.8	98.6	98	97.6 ± 1.2
0	98.2	88.4	90.8	97	97.4	94.4 ± 4.4
0.02	98.3	84	80.3	92.7	95.8	90.2 ± 7.7
0.04	66.8	69.8	79.6	89.8	90.3	79.3 ± 10.9
0.06	48.8	52.1	71.1	82.4	80.8	67.0 ± 15.8
0.08	42.4	45	69.7	78	74.9	62.0 ± 17.0

As illustrated in Table 5, the gamma analysis between the TPS‐calculated and ArcCHECK‐measured doses demonstrated an average GPR of 98.4 ± 1.1%, which complies with the quality control criteria specified in the AAPM Task Group 218 report.

**TABLE 5:** Gamma pass rates (%) between TPS‐calculated and measured doses for VMAT technique.

Gamma criterion	C‐Easy‐4Arc	C‐Hard‐4Arc	H&N‐3Arc	Multi‐4Arc	Prostate‐2Arc	Average
3%/2 mm	98.9	98.3	96.5	99.1	99.2	98.4 ± 1.1

Based on a comprehensive triangular gamma analysis above, for VMAT plans pre‐screened with TG‐119 test cases, ARLFO = −0.04 cm (−0.4 mm) and −0.02 cm (−0.2 mm) were identified as the optimal candidate parameter values.

#### 3.1.2 IMRT results

**FIGURE 4:**
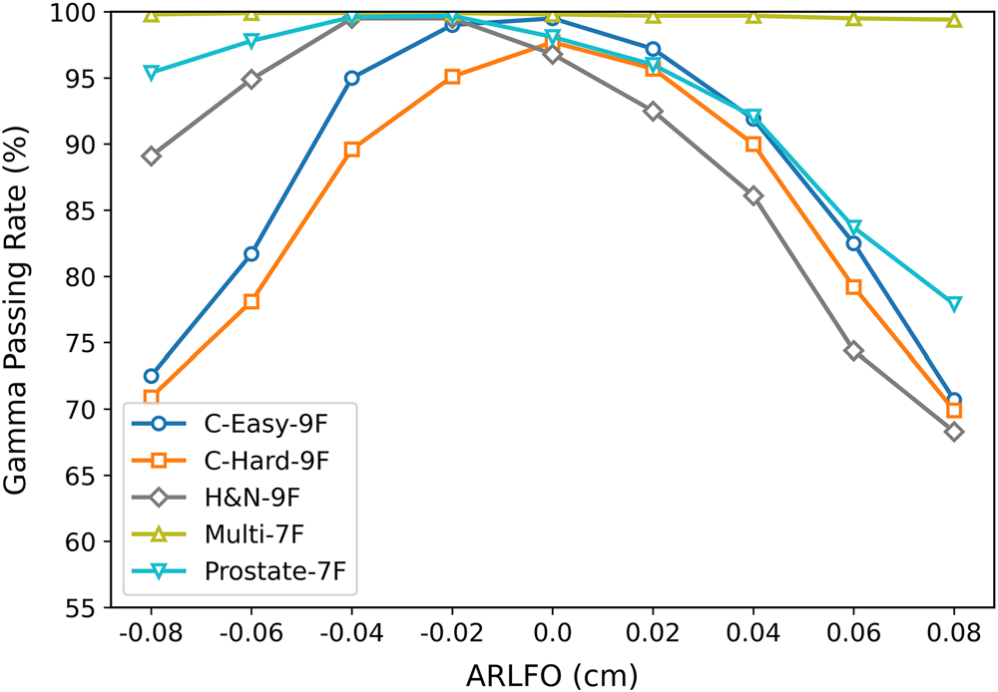
Gamma pass rates (%) between RadCalc Monte Carlo‐simulated doses and TPS‐calculated doses corresponding to different ARLFO values in IMRT technique for TG‐119 test cases.

Our gamma analysis of dose distributions between RadCalc‐calculated and TPS‐calculated data revealed significantly distinct responses to ARLFO variations between the IMRT and VMAT techniques. As shown in Figure 4 and Table 6, For ARLFO < 0, IMRT exhibited greater sensitivity to ARLFO reduction than VMAT, with GPR decreasing as ARLFO decreased. For ARLFO > 0 cm, both techniques showed a monotonic GPR decline, with IMRT demonstrating a more pronounced deterioration as the ARLFO increased. This heightened sensitivity of IMRT plans is consistent with their inherently higher modulation complexity. As quantified in Table 3, the averaged‐MCS values for the IMRT plans (ranging from 0.03 to 0.13) were substantially lower than those for the VMAT plans (ranging from 0.07 to 0.29), confirming that the IMRT plans employed in this study were globally more complex. This reinforces the conclusion that higher modulation complexity leads to greater susceptibility to inaccuracies in the MLC model parameter, with IMRT techniques being particularly affected. Through weighted averaging, we identified ARLFO = −0.04 cm (−0.4 mm), −0.02 cm (−0.2 mm), and 0 cm (0 mm) as the optimal candidate parameter values.

**TABLE 6:** Gamma pass rates (%) between RadCalc and TPS‐calculated doses for TG‐119 test cases using IMRT.

ARLFO (cm)	C‐Easy‐9F	C‐Hard‐9F	H&N‐9F	Multi‐7F	Prostate‐7F	Average
−0.08	72.5	70.9	89.1	99.8	95.4	85.6 ± 13.2
−0.06	81.7	78.1	94.9	99.9	97.8	90.5 ± 9.9
−0.04	95.0	89.6	99.5	99.9	99.6	96.7 ± 4.5
−0.02	99.0	95.1	99.5	99.9	99.7	98.7 ± 2.0
0	99.5	97.7	96.8	99.8	98.1	98.4 ± 1.3
0.02	97.2	95.7	92.5	99.7	96.0	96.2 ± 2.6
0.04	91.9	90.0	86.1	99.7	92.1	92.0 ± 5.0
0.06	82.5	79.2	74.4	99.5	83.7	83.8 ± 9.5
0.08	70.7	69.9	68.3	99.4	77.9	77.2 ± 12.9

Abbreviation: F = Field.

**FIGURE 5:**
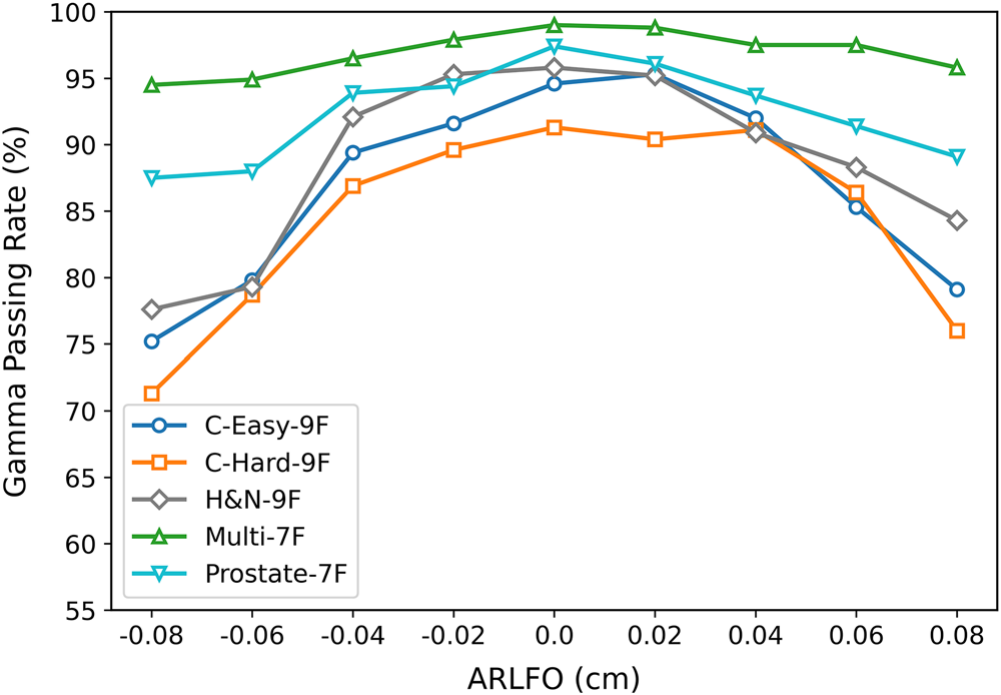
Gamma pass rates (%) between RadCalc Monte Carlo‐simulated doses and ArcCHECK‐measured doses corresponding to different ARLFO values in IMRT technique for TG‐119 test cases.

Our gamma analysis of dose distributions between RadCalc‐calculated and ArcCHECK‐measured data revealed a non‐monotonic relationship between ARLFO and the weighted average GPR. As illustrated in Figure 5 and Table 7, when ARLFO increased from −0.08 cm, the weighted average GPR initially increases and then decreases, peaking at ARLFO = 0 cm (0 mm). Through weighted averaging, we identified ARLFO = −0.02 cm (−0.2 mm), 0 cm (0 mm), and 0.02 cm (0.2 mm) as the optimal candidate parameter values.

**TABLE 7:** Gamma pass rates (%) between RadCalc and ArcCHECK‐measured doses for TG‐119 test cases using IMRT.

ARLFO (cm)	C‐Easy‐9F	C‐Hard‐9F	H&N‐9F	Multi‐7F	Prostate‐7F	Average
−0.08	75.2	71.3	77.6	94.5	87.5	81.2 ± 9.5
−0.06	79.8	78.7	79.3	94.9	88	84.1 ± 7.1
−0.04	89.4	86.9	92.1	96.5	93.9	91.8 ± 3.8
−0.02	91.6	89.6	95.3	97.9	94.4	93.8 ± 3.2
0	94.6	91.3	95.8	99	97.4	95.6 ± 2.9
0.02	95.3	90.4	95.2	98.8	96.1	95.2 ± 3.0
0.04	92	91.1	90.9	97.5	93.7	93.0 ± 2.7
0.06	85.3	86.4	88.3	97.5	91.4	89.8 ± 4.9
0.08	79.1	76	84.3	95.8	89.1	84.9 ± 7.9

Abbreviation: F = Field.

The gamma analysis results comparing the TPS‐calculated and TPS‐measured doses are summarized in Table 8. The GPR was 97.6% ± 2.6%, meeting the criteria specified in AAPM Task Group Report 218.

**TABLE 8:** Gamma pass rates (%) between TPS‐Calculated and measured doses for IMRT technique.

Gamma criterion	C‐Easy‐9F	C‐Hard‐9F	H&N‐9F	Multi‐7F	Prostate‐7F	Average
3%/2 mm	98.3	93.1	98.1	100	98.7	97.6 ± 2.6

Abbreviation: F = Field.

For the IMRT technique, the preliminary screening conclusions derived from the RadCalc versus TPS and RadCalc versus ArcCHECK‐measured doses were not entirely consistent. Therefore, we selected the overlapping parameter values from both conclusions, namely ARLFO = −0.02 cm (−0.2 mm) and 0 cm (0 mm), as the optimal candidate parameter values.

### 3.2 Benchmark test cases

#### 3.2.1 VMAT results

The gamma analysis results for the benchmark test cases are listed in Table 9. When ARLFO was set to −0.02 cm, the GPR was consistently higher in both RadCalc‐calculated doses versus ArcCHECK‐measured doses and RadCalc‐calculated versus TPS‐calculated doses, indicating −0.02 cm as the optimal value. Additionally, the average GPR between ArcCHECK‐measured doses and TPS‐calculated doses reached 99.7% ± 0.4%, demonstrating high consistency among all three dose distributions at ARLFO = −0.02 cm (−0.2 mm). Consequently, for the VMAT technique, the ARLFO parameter was finalized at −0.02 cm (−0.2 mm) through benchmark fine‐tuning and applied to subsequent clinical validation.

**TABLE 9:** Gamma pass rates (%) of triangle gamma analysis of benchmark cases (VMAT and IMRT).

Technique	RadCalc vs TPS			RadCalc vs Measurement			Measurement vs TPS
	A = 0	A = −0.02	A = −0.04	A = 0	A = −0.02	A = −0.04	–
VMAT	–	98.7 ± 1.1	97.6 ± 1.8	–	99.1 ± 0.7	98.6 ± 1.2	99.7 ± 0.4
IMRT	96.4 ± 2.3	97.5 ± 1.7	–	97.4 ± 1.1	95.5 ± 2.0	–	99.4 ± 0.6

Abbreviation: A = ARLFO.

#### 3.2.2 IMRT results

Table 10 presents the gamma analysis results for the benchmark test cases. When comparing RadCalc‐calculated doses with TPS‐calculated doses, the GPR was higher at ARLFO = −0.02 cm (−0.2 mm). However, when comparing RadCalc‐calculated doses with ArcCHECK‐measured doses, GPR peaked at ARLFO = 0 cm (0 mm). This discrepancy between the two fine‐tuning conclusions requires further investigation. For the IMRT technique, using ARLFO = 0 cm(0 mm) would necessitate establishing separate modeling and data transfer pathways, increasing the clinical operational risks^29^ Moreover, even at ARLFO = −0.02 cm (−0.2 mm), the average GPR between RadCalc‐calculated doses and ArcCHECK‐measured doses was 95.5% ± 2.0%, still meeting the action threshold of the AAPM Task Group Report 218. Consequently, the ARLFO parameter was uniformly set to −0.02 cm (−0.2 mm) for IMRT through benchmark fine‐tuning and applied to subsequent clinical validation.

**TABLE 10:** Averaged gamma pass rates (%) of triangle gamma analysis of 40 clinical cases when ARLFO = −0.02 cm.

Treatment site	RadCalc vs TPS	RadCalc vs ArcCHECK	ArcCHECK vs TPS
Head and neck	98.9 ± 0.8	97.5 ± 2.8	99.5 ± 0.8
Chest	98.5 ± 0.8	95.6 ± 2.8	99.2 ± 1.0
Abdomen	99.6 ± 0.3	96.3 ± 2.9	99.5 ± 0.9
Pelvis	99.1 ± 0.5	99.4 ± 0.5	99.9 ± 0.2

### 3.3 Clinical test cases

The triangular gamma analysis results for the 40 clinical cases (10 head, 10 thorax, 10 abdomen, and 10 pelvis) are summarized in Table 10. All GPR exceeded 95%, satisfying the quality control criteria of AAPM Task Group Report 218. This indicates that with ARLFO = −0.02 cm (−0.2 mm), RadCalc can effectively perform secondary verification of TPS‐calculated doses while concurrently providing auxiliary checks for ArcCHECK‐based verification plans imported into RadCalc, thereby reducing the workload of patient‐specific quality assurance (PSQA).

## 4 DISCUSSION

The escalating complexity of modern radiotherapy plans significantly amplifies the clinical workload for measurement‐based quality assurance. Independent secondary dose calculation software effectively alleviates this verification burden for patient‐specific QA. Moreover, it serves as a critical element in the ART workflow guided by either MRI or CBCT. However, prior to clinical deployment, such software requires rigorous commissioning and validation. This study adhered to the AAPM TG‐219 guidelines for commission and validation of the Monte Carlo algorithm in RadCalc for the O‐ring linac Halcyon. We utilized the TG‐119 benchmark test cases and custom‐designed IMRT/VMATplans with the corresponding ArcCHECK phantom‐based verification plans. Through triangular gamma analysis comparing the TPS‐calculated, RadCalc‐calculated, and measured doses, we progressively narrowed the ARLFO parameter range to identify its optimal value. Finally, the reliability of the optimized model was evaluated using 40 clinical cases.

The RadCalc software employs a sophisticated Monte Carlo model of the linear accelerator head, which includes a three‐dimensional representation of the MLC leaves based on manufacturer specifications. For complex geometries such as the double‐stack MLC in O‐ring linac, the parameter ARLFO functions as a spatial offset that adjusts the effective position of the MLC leaves in the model. This adjustment is crucial for fine‐tuning the agreement between the calculated and measured radiation fields, particularly near the leaf ends.

The functionality of ARLFO is analogous to the DLG parameter used in TPS like Eclipse. The DLG is a commissioning parameter that accounts for the rounded leaf tip effect and transmission, effectively modifying the gap between opposing leaves in the dose calculation algorithm to match measured data. Similarly, the ARLFO can be conceptualized as a spatial correction that optimizes the MLC model in the independent dose calculation algorithm. While the DLG primarily addresses the dosimetric consequences of the leaf's curvature, the ARLFO directly adjusts the geometric position of the entire 3D leaf model to achieve a similar goal: ensuring that the simulated radiation field edge matches the physical reality. This distinction arises because RadCalc uses a full 3D geometric model of the leaves, whereas many TPS algorithms use a simplified 1D representation, making the DLG a necessary empirical correction. Therefore, both parameters serve as essential tuning factors during commissioning to align their respective calculation engines with measured data.

Our analysis revealed distinct trends between the VMAT and IMRT techniques. In Eclipse v15.6, the VMAT plans consistently utilized 178 control points, typically configured with two or four arcs. In contrast, IMRT plans employ the sliding window technique with 166 control points per field, predominantly designed with seven or nine fields. The total number of control points differed significantly between the IMRT and VMAT groups. Although each control point represents only an instantaneous position along the MLC trajectory rather than a discrete subfield, the higher control point count in IMRT may imply greater susceptibility to modeling artifacts induced by the ARLFO parameter. IMRT plans, exhibiting higher complexity (lower MCS values), demonstrated significantly greater sensitivity to ARLFO variations, as evidenced by steeper declines in gamma passing rates. This was particularly pronounced in TG‐119 test cases, where both C‐shape‐easy and C‐shape‐hard plans showed exceptional sensitivity, especially in multi‐target scenarios. Their inherent complexity—in geometric design, dosimetric objectives, and clinical implementation challenges—makes them excellent stress tests for MLC modeling accuracy. The observed responses confirm that TG‐119 test cases effectively reflect ARLFO's impact on plans of varying complexity, making them highly suitable for commissioning secondary dose calculation software like RadCalc.

According to the AAPM TG‐219 Report, benchmark test cases should be prioritized for validation following any model modification or upgrade. These cases encompass patients with tumors across diverse anatomical sites to maximally replicate the spectrum of clinical scenarios encountered in practice. Given that each institution's accelerator serves distinct patient populations with varying disease distributions and volumes, the quality management physicist (QMP) must strategically select appropriate test cases tailored to local clinical demands.

In clinical validation cases, triangular gamma analysis results consistently demonstrated an average GPR exceeding 97% across head and neck, thoracic, abdominal, and pelvic patients. This confirms that RadCalc accurately performs independent verification of the original plans and provides supplementary validation for ArcCHECK‐based verification plans. The GPR between the RadCalc‐calculated doses and TPS‐calculated doses and ArcCHECK‐measured doses vs. TPS‐calculated doses exceeded 95%, whereas the direct GPR comparison between the RadCalc‐calculated doses and ArcCHECK‐measured doses occasionally approached 90%, indicating a relatively lower agreement. This suggests that relying solely on third‐party independent verification software may lead to a partial underestimation of discrepancies and cannot fully substitute for physical measurements. Additionally, the validation of this study was limited to 40 patient plans, restricting further exploration of model computational accuracy across anatomical sites.

In terms of the analytical methodology, we innovatively implemented triangular gamma analysis to comprehensively evaluate dose verification systems. Comparing RadCalc‐calculated dose distributions with TPS‐calculated distributions replicates the secondary dose verification scenario in clinical practice: after completing treatment planning, medical physicists transfer the patient's CT images, plans, and doses from the TPS to RadCalc for independent verification using its Monte Carlo algorithm. Comparing RadCalc‐calculated doses with ArcCHECK‐measured dose distributions reflects RadCalc's role in streamlining PSQA workflows. Physicists can import verification plans from the TPS into RadCalc for Monte Carlo simulation, significantly reducing the physical measurement workload. Crucially, directly comparing TPS‐calculated and ArcCHECK‐measured dose distributions serves as a benchmark reference to prevent algorithmic bias toward either reference system during the RadCalc commissioning. Although this method requires substantial computational resources for simulations, it ensures the versatility of RadCalc across clinical applications, enhancing the reliability of secondary dose verification while optimizing PSQA efficiency. Consequently, we recommend extending this methodology to commissioning similar secondary dose‐calculation software systems.

While the final commissioning outcome set ARLFO = −0.02 cm(‐0.2 mm) for both VMAT and IMRT techniques, this value likely represents a compromise solution for clinical practicality, driven by the operational need to simplify the clinical workflow and mitigate risks, justified by the fact that it yielded clinically excellent GPR results for all validation pathways, even when it was not the absolute optimum for a single specific comparison. Moreover, this study limited ARLFO adjustments to 9 discrete values (−0.08 cm (−0.8 mm) to +0.08 cm (+0.8 mm)). Although −0.02 cm (−0.2 mm) was identified as the clinically optimal value within this search grid, a future investigation with a refined sweep around this value could potentially yield a more precise optimum.

Our findings are based on the Anisotropic Analytical Algorithm (AAA). It is important to consider that the choice of TPS algorithm may influence the results. For instance, Acuros XB, which uses a deterministic solver for the linear Boltzmann transport equation, might exhibit a different baseline agreement with Monte Carlo calculations compared to AAA. Consequently, while our methodology for determining the optimal ARLFO remains valid, the absolute value of this parameter and the resulting gamma pass rates could be algorithm‐dependent. Institutions using different TPS algorithms should account for this during their local commissioning.

As for planner experience, while not directly quantified in this study, its effect is inherently embedded in the modulation complexity of the clinical plans we used for validation. An experienced planner tends to create efficient, less modulated plans (higher MCS) that are inherently more robust, as demonstrated by our results. The fact that our optimized ARLFO model successfully achieved high gamma pass rates across a diverse set of 40 clinical plans from multiple disease sites suggests that the commissioning process itself accounts for the variation in planning style and complexity typically encountered in clinical practice. In this way, our validation against real‐world clinical cases indirectly incorporates and confirms the model's robustness to the variations introduced by different planners.

The methodological framework established in this study provides a robust foundation for specific commissioning needs beyond standard IMRT/VMAT. First, its applicability to stereotactic body radiation therapy (SBRT) plans warrants consideration, given their more stringent quality assurance constraints. The high modulation and small fields characteristic of SBRT place even greater demands on MLC modeling accuracy, making the optimal tuning of parameters like ARLFO critically important. Second, the efficient, patient‐specific 3D dose verification protocol presents a promising path for ART platforms like Ethos, offering a Monte Carlo‐based alternative for validating daily adaptive plans. Future work will therefore focus on applying and validating this commissioning protocol specifically for SBRT plans and ART workflows to ensure its reliability across all advanced treatment modalities.

## 5 CONCLUSION

In summary, this study demonstrates that commissioning the ARLFO parameter is essential for the 3D Monte Carlo‐based independent dose calculation software RadCalc before clinical deployment. While the optimal ARLFO value of −0.02 cm (−0.2 mm) was determined for our specific Halcyon accelerator, the primary contribution of this work is the establishment of a comprehensive commissioning recipe. This recipe, which utilizes TG‐119 and institutional benchmark cases coupled with triangular gamma analysis, provides a rigorous methodological framework for other clinics to follow. We recommend that each institution perform a similar commissioning process to determine their own optimal ARLFO value, ensuring that the RadCalc model is accurately tuned to their local linear accelerator and clinical practice. This approach guarantees reliable independent plan verification based directly on patient CT images and provides robust auxiliary validation for physical measurements.

## AUTHOR CONTRIBUTIONS

**Xiangyin Meng**: Conceptualization (lead); writing—original draft (lead); Investigation (lead). **Tingting Dong**: Data curation (lead); writing—original draft (lead); methodology (lead). **Yongguang Liang**: Resources (equal); review and editing (equal). **Zhen Zhou**: Formal analysis (equal); investigation (equal). **Zhiqun Wang**: Methodology (equal); investigation (equal). **Jie Qiu**: Project administration (lead); funding acquisition (lead). **Jingjing Zhao**: Formal analysis (equal); software (lead). **Zheqing Zhang**: Resources (equal); supervision (equal). **Wenbo Li**: Supervision (equal); writing—review & editing (equal). **Bo Yang**: Supervision (equal); writing—review & editing (equal).

## CONFLICT OF INTEREST STATEMENT

The authors declare no conflicts of interest.

## ETHICS STATEMENT

All procedures performed in studies involving human participants were in accordance with the ethical standards of the institutional and/or national research committee and with the 1964 Helsinki Declaration and its later amendments or comparable ethical standards. The study was approved by the ethics committee at Peking Union Medical College Hospital.

## Data Availability

Research data are available and can be obtained by contacting the corresponding author.
